# Proton pump inhibitors may hinder hypophosphatemic effect of lanthanum carbonate, but not of ferric citrate hydrate or sucroferric oxyhydroxide, in hemodialysis patients

**DOI:** 10.1080/0886022X.2020.1803085

**Published:** 2020-08-11

**Authors:** Hitoshi Minakuchi, Tadashi Yoshida, Noriko Kaburagi, Teppei Fujino, Sho Endo, Tomoko Yamashita Takemitsu, Norimasa Yamashita, Hiroshi Itoh, Mototsugu Oya

**Affiliations:** a Apheresis and Dialysis Center, Keio University School of Medicine, Tokyo, Japan; b Seigakai Shibuya Station Clinic, Tokyo, Japan; c Seigakai Yoyogi Station Clinic, Tokyo, Japan; d Department of Internal Medicine, Keio University School of Medicine, Tokyo, Japan; e Department of Urology, Keio University School of Medicine, Tokyo, Japan

**Keywords:** Drug interaction, end-stage renal disease, phosphate, phosphate binders, proton pump inhibitors

## Abstract

Because end-stage renal disease patients undergoing hemodialysis frequently take acid suppressants for the treatment or prevention of gastrointestinal diseases, it is important to clarify the drug-interactions between acid suppressants and phosphate binders on the control of serum phosphate levels. In the present study, we examined whether the phosphate-lowering effects of three phosphate binders, lanthanum carbonate (LC), ferric citrate hydrate (FCH), and sucroferric oxyhydroxide (SFOH), were affected by proton pump inhibitors (PPIs) in maintenance hemodialysis patients. Laboratory data for 71 patients who had been newly prescribed one of the three phosphate binders were examined. LC at a dosage of 500 ± 217 mg/day significantly decreased serum phosphate levels by −18% in the absence of a PPI (*n* = 9), while a dosage of 700 ± 230 mg/day only decreased it by −3% in the presence of a PPI (*n* = 10). Thus, the efficacy of LC in reducing serum phosphate levels was significantly hindered by the presence of PPIs. FCH significantly decreased serum phosphate levels by −18% in the absence of a PPI (*n* = 7, FCH: 571 ± 189 mg/day) and by −17% in the presence of a PPI (*n* = 20, FCH: 638 ± 151 mg/day). The decrease in serum phosphate levels by SFOH (393 ± 197 mg/day) was −7% in the absence of a PPI (*n* = 7), and SFOH at a dosage of 556 ± 316 mg/day significantly decreased serum phosphate levels by −13% in the presence of a PPI (*n* = 18). These results suggest that the phosphate-lowering effect of LC, but not of FCH or SFOH, is diminished in the presence of PPIs in hemodialysis patients.

## Introduction

Hyperphosphatemia is associated with increased mortality in end-stage renal disease (ESRD) patients undergoing hemodialysis (HD) [1]. It is a major factor promoting the formation of arterial medial calcification by enhancing the phenotypic switching of vascular smooth muscle cells into osteoblast-like cells [2,3]. Arterial medial calcification results in increased vessel wall stiffness, decreased compliance and elevated systolic blood pressure, leading to an increase in cardiovascular events and mortality [1]. The control of serum phosphate levels is, therefore, critical for the prognosis of HD patients.

For the treatment of hyperphosphatemia, several phosphate binders, including calcium carbonate, sevelamer hydrochloride, bixalomer, lanthanum carbonate (LC), ferric citrate hydrate (FCH) and sucroferric oxyhydroxide (SFOH), are currently available in clinical practice in Japan [4]. Basically, these compounds bind with phosphate in an insoluble form in the gastrointestinal tract, prevent it from being absorbed, and excrete it in the stool. As a result, an elevation in serum phosphate concentrations is hindered. However, the results of previous studies have shown that the inhibitory effect of calcium carbonate on hyperphosphatemia is diminished by the co-prescription of acid suppressants, such as proton pump inhibitors (PPIs) and histamine H2-receptor antagonists, in HD patients [5–8]. Moreover, although controversial, the phosphate binding affinity of multiple phosphate binders, such as calcium carbonate, sevelamer hydrochloride, and LC, has been shown to be pH-dependent in *in vitro* studies [9–12]. Based on the results of these preceding studies, we hypothesized that the co-prescription of acid suppressants may affect the performance of multiple phosphate binders in HD patients.

Because a number of HD patients take acid suppressants for the treatment or prevention of gastrointestinal symptoms and diseases, it is of critical importance to clarify the drug-interactions between acid suppressants and phosphate binders and their effects on the control of serum phosphate levels. In the present study, we examined whether the phosphate-lowering effects of three phosphate binders, LC, FCH, and SFOH, were affected by the presence or absence of PPIs in maintenance HD patients.

## Methods

### Study subjects

**Figure 1.**
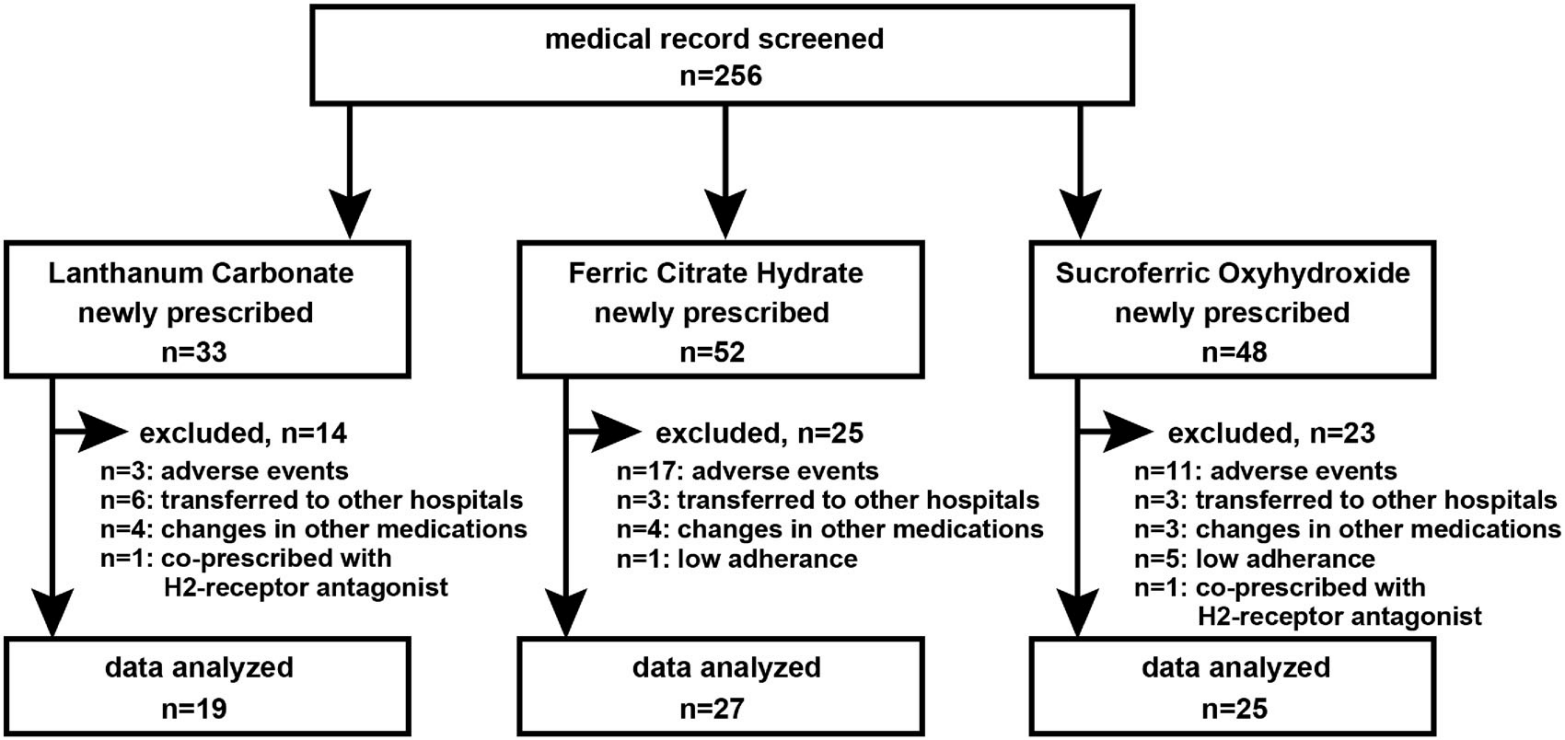
Patient disposition. Adverse events in the LC group were nausea (*n* = 2) and hypophosphatemia (*n* = 1), whereas those in the FCH group were diarrhea (*n* = 8), discolored feces (*n* = 3), constipation (*n* = 2), abdominal discomfort (*n* = 2), nausea (*n* = 1), and liver dysfunction (*n* = 1). Adverse events in the SFOH group included diarrhea (*n* = 6), nausea (*n* = 3), constipation (*n* = 1), and abdominal discomfort (*n* = 1).

This retrospective study was conducted at three dialysis facilities in Japan. The study protocol was approved by the Ethics Committees of Keio University School of Medicine (IRB Approval Number: 20180065), Seigakai Shibuya Station Clinic (IRB Approval Number: 2018S01), and Seigakai Yoyogi Station Clinic (IRB Approval Number: 2018Y01). Written informed consent was obtained from all individual participants at Seigakai Shibuya Station Clinic and Seigakai Yoyogi Station Clinic but was waived for participants at Keio University Hospital. The opportunity to opt out of the study was provided to the participants. The subjects of the present study were ESRD patients who had been undergoing maintenance HD therapy for more than 2 months and who were prescribed one of three phosphate binders (LC, FCH, or SFOH) between 1 January 2016 and 31 December 2017 for the first time. Laboratory data obtained immediately before the prescription and 11–28 days after the prescription were compared. We were able to conduct this study because all PPIs were only available as prescribed medicines in Japan and because all the participants had been educated not to use over-the-counter drugs. Patients whose HD conditions and/or co-treatments for chronic kidney disease-mineral and bone disorder (CKD-MBD) had changed between before and after the prescription examination were excluded from the study. Patients who had been prescribed histamine H2-receptor antagonists were also excluded from the study. Patients who stopped taking the newly prescribed phosphate binder or were transferred to another hospital before the post-prescription examination were excluded from the study. A total of 256 medical records were screened, and we found that 33, 52, and 48 HD patients were newly prescribed with LC, FCH, and SFOH, respectively (Figure 1). However, 14, 25, and 23 patients in each group met the exclusion criteria. Consequently, 19, 27, and 25 HD patients in each group were analyzed.

### Data

Data regarding age, sex, weight, body mass index, HD vintage, duration of the HD session, Kt/V, normalized protein catabolic rate (nPCR), ESRD etiology, date and amount of prescription for phosphate binders, presence or absence of a prescription for PPIs and histamine H2-receptor antagonists, presence or absence of co-treatment for CKD-MBD using other phosphate binders, vitamin D analogs and/or calcimimetics, gastrofiberscopy findings before the start of PPI treatment, and the reasons for a PPI prescription were obtained from the patients’ medical records. Kt/V was assessed by the single-pooled urea kinetic model [13]. The nPCR was calculated using Shinzato’s formula [13]. Biochemical data, including the levels of hemoglobin, albumin, urea nitrogen, creatinine, potassium, calcium, phosphate, and intact parathyroid hormone before and/or after the prescription, were obtained.

### Statistical analyses

Normally distributed continuous variables were expressed as the mean ± standard deviation (SD), non-normal variables as the median and interquartile range (IQR), and categorized data as the percentage frequency. To test the differences between groups, a paired *t*-test, Student’s unpaired *t*-test, or one-way ANOVA with a *post-hoc* Fisher’s protected least significant difference test was used for the normally distributed variables, the Mann–Whitney rank-sum test or the Kruskal–Wallis one-way ANOVA on ranks with the Dunn’s *post-hoc* test for non-normal variables, and a χ^2^ test or Fisher’s exact test for categorical data. All the statistical analyses were performed using SigmaPlot/SigmaStat 9 (Systat Software Inc, San Jose, CA). *p* < 0.05 were considered significant.

## Results

### LC, FCH and SFOH each decreased serum phosphate levels significantly in HD patients

**Figure 2.**
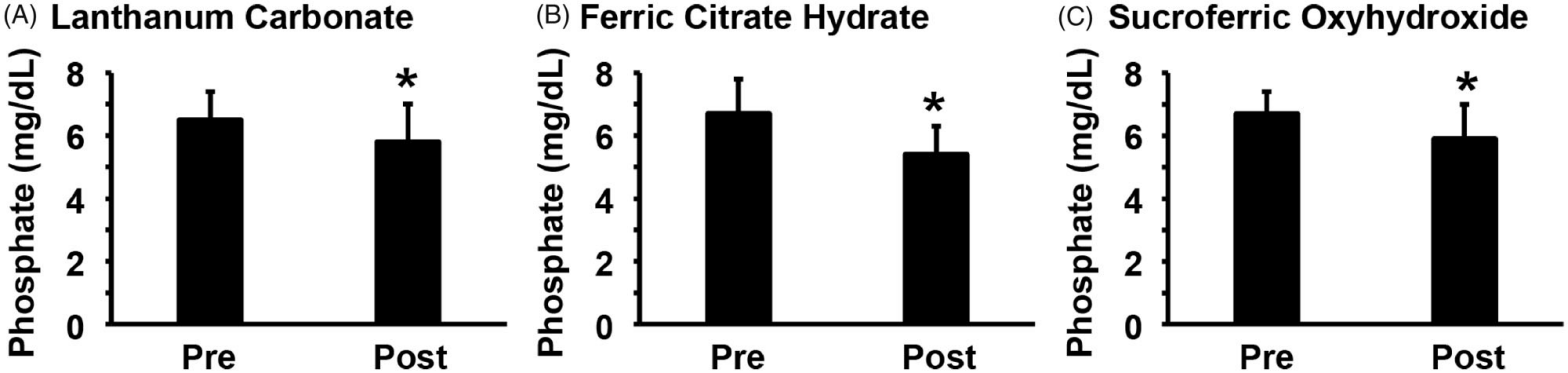
Serum phosphate levels were decreased by phosphate binders in HD patients. HD patients were newly prescribed with LC (A, *n* = 19), FCH (B, *n* = 27), or SFOH (C, *n* = 25). Serum phosphate levels were examined before (Pre) and after (Post) the prescription. Paired *t*-test was performed. *p < 0.05, compared with pre-prescription levels.

Between 1 January 2016 and 31 December 2017, a total of 133 HD patients were newly prescribed with either LC (*n* = 33), FCH (*n* = 52) or SFOH (*n* = 48) for the treatment of hyperphosphatemia. Among them, 19 patients who were prescribed LC, 27 patients who were prescribed FCH, and 25 patients who were prescribed SFOH were analyzed in the present study; the remaining patients met the exclusion criteria and were excluded (Figure 1). The efficacy of phosphate binders was examined by comparing pre-prescription data and post-prescription data examined 11–28 days after the prescription. Age, body weight, body mass index, HD vintage, duration of the HD session, Kt/V, nPCR, etiology of ESRD, and co-treatment for CKD-MBD with phosphate binders, vitamin D analogs or calcimimetics did not differ significantly among the three groups, although the proportion of male patients was higher in patients treated with FCH (Table 1). The biochemical data were not significantly different among the three groups except for the level of urea nitrogen, which was lower in the patients who were prescribed FCH (Table 2). The prescription dosages were 605 ± 240 mg/day for LC, 620 ± 161 mg/day for FCH, and 510 ± 293 mg/day for SFOH. As shown in Figure 2, each of the phosphate binders, including LC, FCH, and SFOH, decreased serum phosphate levels significantly. Indeed, serum phosphate levels were decreased from 6.5 ± 0.9 mg/dL to 5.8 ± 1.2 mg/dL by LC, from 6.7 ± 1.1 mg/dL to 5.4 ± 0.9 mg/dL by FCH, and from 6.7 ± 0.7 mg/dL to 5.9 ± 1.1 mg/dL by SFOH. These results suggest that phosphate binders decrease serum phosphate levels efficaciously in HD patients.

**Table 1.** Patient characteristics.

	LC (*n* = 19)	FCH (*n* = 27)	SFOH (*n* = 25)	*p-*value
Age (years)	60 (56–66)	62 (51–68)	64 (56–69)	0.530^b^
Male (%)	47	85	40	0.002^c^
Body weight (kg)	57 (49–66)	60 (53–74)	53 (48–66)	0.076^b^
Body mass index (kg/m^2^)	22.4 (20.0–25.0)	22.2 (20.0–25.4)	21.0 (19.0–24.2)	0.420^b^
HD vintage (months)	78 (51–259)	82 (56–157)	116 (38–323)	0.722^b^
Duration of HD session (hours)	4 (4–4)	4 (4–4)	4 (4–4)	0.120^b^
Kt/V	1.52 ± 0.28	1.50 ± 0.26	1.49 ± 0.27	0.229^a^
nPCR (g/kg/day)	1.1 ± 0.2	1.0 ± 0.2	1.1 ± 0.3	0.116^a^
ESRD etiology (%)				0.497^c^
Glomerulonephritis	37	48	44	
Diabetes	26	22	20	
Hypertension	11	7	12	
Polycystic kidney disease	0	15	4	
Others	26	7	20	
Co-treatment of CKD-MBD (%)				
Calcium carbonate	58	56	60	0.949^c^
Sevelamer or bixalomer	16	22	12	0.610^d^
LC	–	56	52	0.983^c^
FCH or SFOH	37	–	–	–
Vitamin D analogues	84	96	76	0.106^d^
Calcimimetics	53	33	44	0.417^c^
Duration from prescription to post-examination (days)	19 (12–25)	12 (12–18)	17 (12–20)	0.178^b^

^a^One-way ANOVA test.

^b^Kruskal–Wallis one-way ANOVA on Ranks test.

^c^χ^2^ test.

^d^Fisher’s exact test.

**Table 2.** Laboratory data before the prescription of phosphate binders.

	LC (*n* = 19)	FCH (*n* = 27)	SFOH (*n* = 25)	*p*-value
Hemoglobin (g/dl)	11.4 ± 0.7	11.1 ± 0.8	11.1 ± 0.6	0.279^a^
Albumin (g/dl)	3.6 (3.5–3.8)	3.7 (3.5–3.8)	3.6 (3.3–3.7)	0.825^b^
Urea nitrogen (mg/dl)	73 ± 12	63 ± 12	71 ± 14	0.030^a^
Creatinine (mg/dl)	11.0 ± 2.5	12.2 ± 2.7	11.2 ± 2.7	0.225^a^
Calcium (mg/dl)	8.8 ± 0.6	8.9 ± 0.5	8.7 ± 0.6	0.537^a^
Phosphate (mg/dl)	6.5 ± 0.9	6.7 ± 1.1	6.7 ± 0.7	0.755^a^
Parathyroid hormone (pg/ml)	268 (192–337)	207 (141–268)	288 (195–447)	0.097^b^
Phosphate, average (mg/dl)	5.9 ± 0.7	5.9 ± 0.7	6.2 ± 0.5	0.199^a^

Uncorrected serum calcium levels are shown. The phosphate average means the average of pre-dialysis serum phosphate levels during 3 months before the prescription of the phosphate binder.

^a^One-way ANOVA with a *post-hoc* Fisher’s protected least significant difference test.

^b^Kruskal–Wallis one-way ANOVA on Ranks test.

### PPIs hindered the phosphate-lowering effect of LC, but not of FCH or SFOH, in HD patients

We next examined whether the phosphate-lowering effects of LC, FCH, and SFOH were affected by the presence of PPIs. The characteristics of the patients enrolled in the present study were re-analyzed according to the presence or absence of a prescription for PPIs (Table 3). Most of the characteristics, including the gastrofiberscopy findings, were not different among the groups. As exceptions, HD vintage, duration of the HD session, and Kt/V differed significantly between the patients who were prescribed SFOH and a PPI and those who were prescribed SFOH without a PPI. In addition, the patients who were prescribed SFOH and a PPI were co-treated with LC more frequently (67%) than the patients who were prescribed SFOH without a PPI (14%). However, a multiple regression analysis showed no association between the decrease in serum phosphate levels and the concomitant use of phosphate binders (data not shown). Laboratory data did not differ among the groups, except for serum albumin levels in the patients who were prescribed SFOH and a PPI versus those who were prescribed SFOH without a PPI (Table 4).

**Table 3.** Characteristics of patients newly prescribed a phosphate binder, stratified according to the presence or absence of a PPI.

	LC			FCH			SFOH		
	Without PPI (*n* = 9)	With PPI (*n* = 10)	*p*-value	Without PPI (*n* = 7)	With PPI (*n* = 20)	*p*-value	Without PPI (*n* = 7)	With PPI (*n* = 18)	*p-*value
Age (years)	60 ± 5	61 ± 7	0.776^a^	58 ± 13	62 ± 11	0.470^a^	61 ± 9	64 ± 9	0.535^a^
Male (%)	56	40	0.656^d^	86	85	1.000^d^	71	28	0.075^d^
Body weight (kg)	62 ± 10	54 ± 17	0.236^a^	65 ± 17	66 ± 16	0.846^a^	55 (51–70)	51 (47–66)	0.318^b^
Body mass index (kg/m^2^)	23.7 ± 3.6	21.1 ± 4.2	0.176^a^	23.7 ± 5.6	23.2 ± 3.9	0.786^a^	22.2 ± 5.3	22.1 ± 4.6	0.941^a^
HD vintage (months)	111 (51–362)	73 (48–133)	0.438^b^	62 (36–106)	97 (59–167)	0.234^b^	67 ± 128	224 ± 153	0.025^a^
Duration of HD session (hours)	4 (4–4)	4 (4–4)	0.967^b^	4 (4–4)	4 (4–4)	0.451^b^	4 (3–4)	4 (4–4)	0.044^b^
Kt/V	1.46 ± 0.18	1.58 ± 0.34	0.356^a^	1.44 (1.38–1.47)	1.41 (1.32–1.49)	0.846^b^	1.27 ± 0.16	1.58 ± 0.26	0.008^a^
nPCR (g/kg/day)	1.1 (0.9–1.3)	1.0 (0.9–1.3)	1.000^b^	1.1 ± 0.3	1.0 ± 0.2	0.296^a^	1.1 ± 0.3	1.1 ± 0.2	0.849^a^
ESRD etiology (%)			0.057^c^			0.401^c^			0.686^c^
Glomerulonephritis	67	10		71	40		43	44	
Diabetes	22	30		14	25		29	17	
Hypertension	0	20		0	10		0	17	
Polycystic kidney disease	0	0		0	20		0	6	
Others	11	40		14	5		29	17	
Co-treatment of CKD-MBD (%)									
Calcium carbonate	67	50	0.650^d^	57	61	1.000^d^	86	50	0.179^d^
Sevelamer or bixalomer	22	10	0.582^d^	14	25	1.000^d^	29	6	0.180^d^
LC	–	–		43	60	0.662^d^	14	67	0.030^d^
FCH or SFOH	22	50	0.350^d^	–	–		–	–	
Vitamin D analogues	89	80	1.000^d^	100	95	1.000^d^	71	78	1.000^d^
Calcimimetics	56	50	1.000^d^	14	40	0.363^d^	14	56	0.090^d^
Prescribed amount (mg/day)	500 ± 217	700 ± 230	0.068^a^	500 (500–750)	750 (500–750)	0.453^b^	250 (250–500)	500 (250–750)	0.237^b^
Duration from prescription to post-examination (days)	18.2 ± 6.7	18.5 ± 5.9	0.924^a^	12 (12–12)	13 (12–20)	0.437^b^	15.3 ± 4.6	17.2 ± 4.4	0.322^a^
Gastrofiberscopy findings (%)			0.150^c^			0.123^c^			0.056^c^
Normal	0	20		0	15		0	11	
Reflux esophagitis	22	20		0	25		0	33	
Gastritis	44	30		57	45		43	44	
Gastric ulcer	0	20		0	5		0	0	
Gastric cancer	0	10		0	5		0	0	
Not performed	33	0		43	5		57	11	
Reasons of PPI prescription (%)									
Reflux esophagitis	–	10		–	30		–	28	
Gastric lesions	–	70		–	65		–	39	
Prevention of gastric bleeding	–	40		–	25		–	39	
Unspecified	–	20		–	10		–	11	
Name of PPI prescribed (%)									
Omeprazole	–	30		–	5		–	28	
Lansoprazole	–	10		–	35		–	22	
Rabeprazole	–	30		–	20		–	22	
Esomeprazole	–	20		–	40		–	22	
Vonoprazan	–	10		–	0		–	6	

^a^Unpaired *t*-test.

^b^Mann–Whitney rank-sum test.

^c^χ^2^ test.

^d^Fisher’s exact test.

**Table 4.** Laboratory data before the prescription of phosphate binders, stratified according to the presence or absence of a PPI.

	LC			FCH			SFOH		
	Without PPI (*n* = 9)	With PPI (*n* = 10)	*p-*value	Without PPI (*n* = 7)	With PPI (*n* = 20)	*p*-value	Without PPI (*n* = 7)	With PPI (*n* = 18)	*p*-value
Hemoglobin (g/dl)	11.2 ± 0.5	11.6 ± 0.9	0.300^a^	11.2 ± 0.8	11.0 ± 0.9	0.713^a^	11.2 ± 0.7	11.1 ± 0.7	0.553^a^
Albumin (g/dl)	3.6 ± 0.3	3.6 ± 0.3	0.928^a^	3.9 (3.7–3.9)	3.6 (3.4–3.7)	0.127^b^	3.8 ± 0.2	3.5 ± 0.2	0.006^a^
Urea nitrogen (mg/dl)	78 ± 11	69 ± 11	0.079^a^	68 ± 9	62 ± 13	0.259^a^	72 ± 15	70 ± 14	0.766^a^
Creatinine (mg/dl)	12.0 ± 2.6	10.1 ± 2.1	0.098^a^	12.3 ± 2.8	12.2 ± 2.7	0.905^a^	12.2 (12.0–15.0)	10.7 (9.6–11.5)	0.065^b^
Calcium (mg/dl)	9.1 (8.2–9.7)	8.6 (8.3–8.9)	0.462^b^	8.8 (8.7–9.6)	8.9 (8.7–9.3)	0.698^b^	8.5 ± 0.6	8.8 ± 0.6	0.296^a^
Phosphate (mg/dl)	6.7 ± 0.9	6.3 ± 1.0	0.341^a^	6.4 ± 0.8	6.8 ± 1.2	0.432^a^	6.6 ± 0.4	6.7 ± 0.7	0.550^a^
Parathyroid hormone (pg/ml)	253 ± 101	281 ± 171	0.665^a^	182 ± 90	250 ± 136	0.238^a^	426 (305–545)	257 (169–359)	0.053^b^
Phosphate, average (mg/dl)	6.1 ± 0.7	5.7 ± 0.8	0.330^a^	5.7 (5.4–5.8)	6.0 (5.7–6.4)	0.056^b^	6.2 ± 0.4	6.2 ± 0.6	0.894^a^

Uncorrected serum calcium levels are shown. The phosphate average means the average of pre-dialysis serum phosphate levels during the 3 months before the prescription of the phosphate binder.

^a^Unpaired *t*-test.

^b^Mann–Whitney rank-sum test.

**Figure 3.**
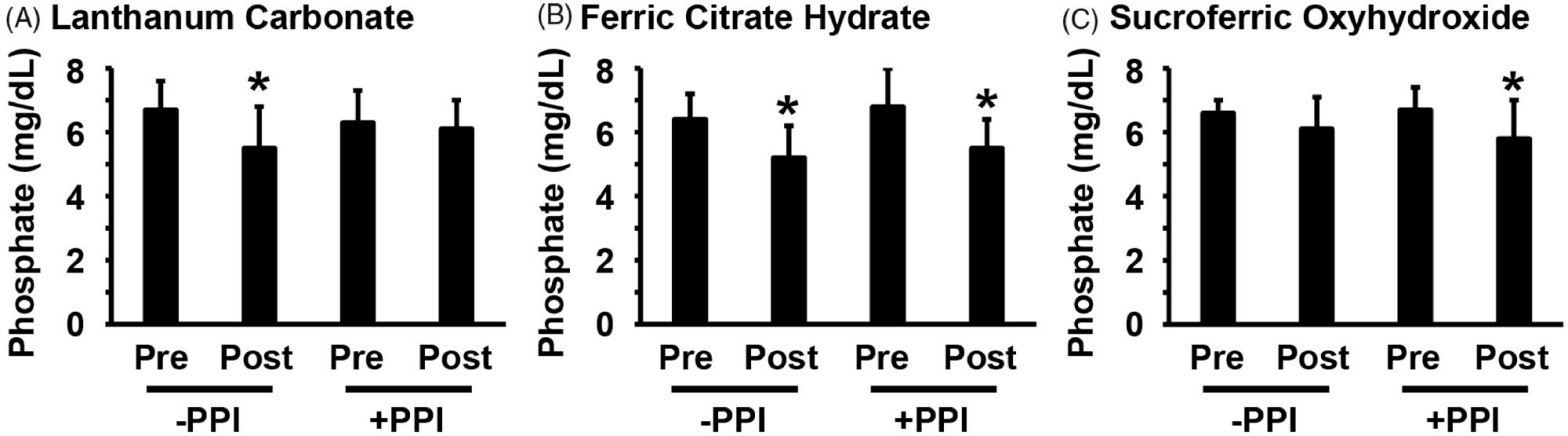
Phosphate-lowering effect of LC, but not of FCH or SFOH, was diminished by PPIs in HD patients. The changes in serum phosphate levels of the HD patients shown in Figure 2 were re-analyzed according to the presence (+PPI) or absence (-PPI) of a prescription for a PPI. One-way ANOVA with a *post-hoc* Fisher’s protected least significant difference test was performed. *p < 0.05, compared with pre-prescription levels.

**Figure 4.**
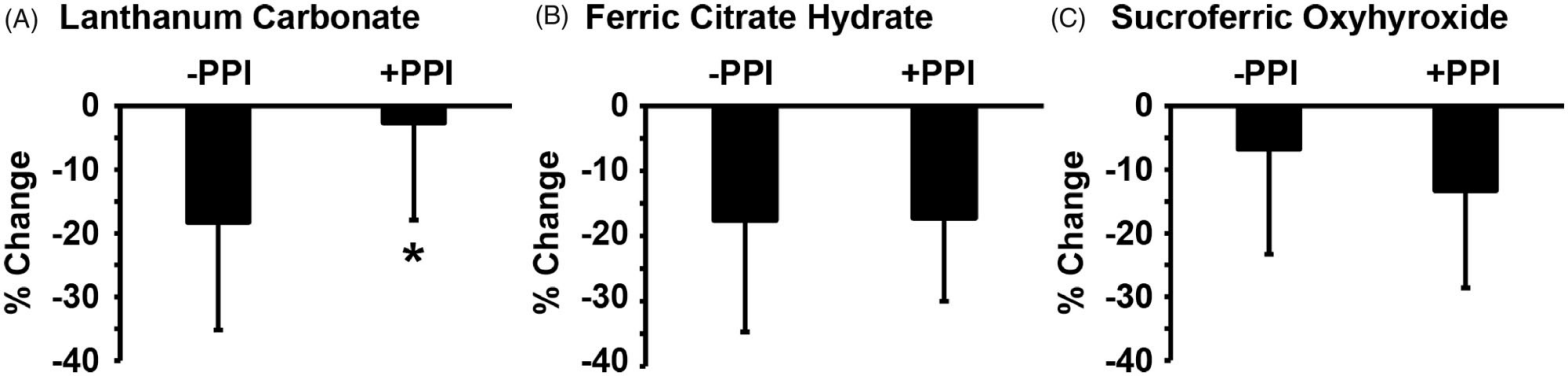
PPIs affected the efficacy of the reduction in serum phosphate levels by LC, but not by FCH or SFOH. Changes in serum phosphate levels by LC (A), FCH (B) or SFOH (C) were compared between HD patients taking a PPI (+PPI) and those who were not taking a PPI (-PPI). Student’s unpaired *t*-test was performed. *p < 0.05, compared with HD patients who were not taking a PPI.

Of significant interest, the phosphate-lowering effect of LC was influenced by the co-prescription of PPIs. As shown in Figure 3(A), LC at a dosage of 500 ± 217 mg/day significantly decreased serum phosphate levels from 6.7 ± 0.9 mg/dL to 5.5 ± 1.3 mg/dL in the absence of a PPI (*n* = 9). By contrast, LC at a dosage of 700 ± 230 mg/day only decreased serum phosphate levels from 6.3 ± 1.0 mg/dL to 6.1 ± 0.9 mg/dL in the presence of a PPI (*n* = 10). The efficacy of LC in reducing serum phosphate levels was significantly hindered by the presence of PPIs (Figure 4(A)). FCH significantly decreased serum phosphate levels from 6.4 ± 0.8 mg/dL to 5.2 ± 1.0 mg/dL in the absence of a PPI (*n* = 7), and from 6.8 ± 1.2 mg/dL to 5.5 ± 0.9 mg/dL in the presence of a PPI (*n* = 20) (Figure 3(B)). The phosphate-lowering effect of FCH was independent from the presence of PPIs (Figure 4(B)). Serum phosphate levels were significantly decreased by SFOH from 6.7 ± 0.7 mg/dL to 5.8 ± 1.2 mg/dL in the presence of a PPI (*n* = 18), whereas SFOH-induced reduction in serum phosphate levels from 6.6 ± 0.4 mg/dL to 6.1 ± 1.0 mg/dL (*n* = 7) was not significant in the absence of a PPI (Figure 3(C)). The efficacy of SFOH in reducing serum phosphate levels was not statistically different between the presence versus absence of PPIs (Figure 4(C)). These results suggest that PPIs hinder the phosphate-lowering effect of LC, but not of FCH or SFOH, in HD patients.

## Discussion

The results of the present study showed that the phosphate-lowering effect of LC, but not of FCH or SFOH, was diminished by PPIs in HD patients. Indeed, LC at a dosage of 500 ± 217 mg/day decreased serum phosphate concentrations by −18% in the absence of PPIs, while a dosage of 700 ± 230 mg/day only decreased serum phosphate concentrations by −3% in the presence of PPIs. By contrast, FCH and SFOH both decreased serum phosphate levels, irrespective of the presence of PPIs. These results are of critical importance, because HD patients frequently suffer from hyperphosphatemia and are treated with phosphate binders and because they often take PPIs as acid suppressants. PPIs are prescribed to a variety of HD patients who require anti-platelet and/or anti-coagulant therapies, who take non-steroidal anti-inflammatory drugs for pain relief, or who have reflux esophagitis and gastroduodenal ulcer. As such, both phosphate binders and PPIs are widely used in HD patients. Thus, clarification of the drug-interaction between LC and PPIs will provide useful information for nephrologists and medical teams at dialysis facilities.

In the present study, we showed that the efficacy of LC in reducing serum phosphate levels was significantly influenced by PPIs in HD patients. Consistent with this finding, the results of previous studies also suggest that the effect of LC is modified by the gastric environment [12,14]. Schumacher et al. [12] reported that, in an *in vitro* setting, the phosphate-binding capacity of LC was dependent on pH, whereas pH had only a slight influence on the phosphate binding of SFOH and calcium carbonate. They showed that LC bound considerably more phosphate in a solution at pH 3.0 than in a solution at pH 6.0 [12]. In addition, Coppolino et al. [14] showed that LC dissolved over a longer time, compared with sevelamer hydrochloride *in vitro*. The slower rate of dissolution and the lower binding affinity may be reasons why LC exhibited a modest effect on serum phosphate concentrations in the presence of PPIs. However, data regarding the intragastric pH of the patients were not available in the present study. It is possible that PPIs hindered the phosphate-lowering effect of LC through mechanisms other than the neutralization of gastric pH, although PPI-induced changes in intragastric pH are likely to be a major factor affecting the efficacy of LC to control serum phosphate concentrations. Further studies are required to address the detailed mechanisms of the drug-interaction between LC and PPIs.

Moreover, the phosphate-binding affinity of a crushed tablet of LC has been shown to be higher than that of a chewable tablet of LC in *in vitro* studies [15]. The difference in dosage forms may have a substantial effect on serum phosphate levels. However, the dosage form of LC prescribed to patients in the present study was either a chewable tablet or an oral powder, irrespective of the presence or absence of PPIs. It is unlikely that the dosage form of LC was a major determinant of the difference in the phosphate-lowering effect of LC between the LC with PPI group and the LC without PPI group. Recently, LC oral disintegrant has become available in clinical practice. Determining to what extent the dosage forms of LC affect the phosphate binding capacity in humans *in vivo* should be a future topic of study.

Of interest, the degree of SFOH-induced reduction in serum phosphate levels tended to be larger in the presence of PPIs than in the absence of PPIs in the present study. This phenomenon was likely to have been caused by differences in SFOH dosages. As shown in Table 4, the prescribed dosage of SFOH was smaller in the patients treated without a PPI (SFOH: 393 ± 197 mg/day), compared with the patients who were treated with a PPI (SFOH: 556 ± 316 mg/day). Because diarrhea had been known as an adverse effect of SFOH [16], some patients began by taking a minimal dose of SFOH. In addition, differences in HD vintage, duration of the HD session, or Kt/V also might have affected the degree of SFOH-induced reduction in serum phosphate concentrations. In the SFOH-treated groups, the patients who were prescribed a PPI had a longer HD vintage, a longer duration of HD session, and a larger Kt/V than the patients who were not prescribed a PPI. Further prospective studies in which the amounts of phosphate binders and the HD conditions are comparable among the groups are required to evaluate the efficacy of phosphate binders accurately.

Nausea and vomiting are known adverse effects of LC [17], whereas diarrhea and constipation are major adverse effects of FCH and SFOH [16,18]. Phosphorus absorption might be affected by these adverse effects. However, as shown in Figure 1, patients who reported these adverse effects stopped taking phosphate binders and were subsequently excluded from the study. In addition, the use of PPIs might alter the amount of food intake in HD patients. This could be a potential bias of the present study, and we thus examined the nPCR, as an indicator of protein intake. The nPCR was not different among the groups. Moreover, we showed that the average of pre-dialysis serum phosphate concentrations during the 3 months before the prescription of phosphate binders did not differ among the groups. These results suggest that food intake was similar among the groups. Nevertheless, we need to pay attention to these points when further studies are planned.

In this study, we compared serum phosphate levels before and after the prescription of new phosphate binders in maintenance HD patients. We could not compare serum phosphate levels before and after the use of PPIs. The latter is also an interesting experimental protocol. However, the recruitment of participants may be difficult, because most HD patients begin taking a PPI before reaching ESRD. In addition, we could not determine the long-term effects of phosphate binders in the presence or absence of PPIs. Indeed, we evaluated the effect of newly prescribed phosphate binders on serum phosphate levels 11–28 days after the start of the prescription. Because this study was retrospective, the dosages of phosphate binders were not consistent over time in many cases. To resolve these points, prospective and interventional studies are required.

This study had several limitations, including a potential selection bias caused by the retrospective nature of the analysis and the relatively small number of Japanese patients included. Particularly, the number of patients who were not treated with a PPI was small in the present study. Moreover, serum phosphate levels are influenced by multiple factors, including changes in diet and drug adherence. Unfortunately, this retrospective study did not evaluate changes in dietary phosphorus intake after the prescription of phosphate binders or drug adherence during the study period. However, all the patients who were evaluated in the present study had equally received counseling regarding a reduction in dietary phosphorus intake so as to enhance adherence to the phosphate binders. In addition, we did not exclude patients who had been treated with multiple phosphate binders. Indeed, calcium carbonate is a neutralizing agent for gastric acid, and it might also affect the phosphate-lowering effects of other phosphate binders. Fortunately, the frequency of the prescription of calcium carbonate was similar between the patients who were prescribed a PPI and non-PPI users in each of the LC, FCH, and SFOH groups. Further studies, such as interventional, crossover, double-blind, randomized, and placebo-controlled trials, are warranted to confirm and extend our findings.

In summary, the results of the present study suggest that the phosphate-lowering effect of LC, but not of FCH or SFOH, is diminished by the presence of PPIs in HD patients. The control of serum phosphate levels is of critical importance for prolonging the life expectancy of ESRD patients. Physicians should be aware of drug interactions between phosphate binders and PPIs to manage CKD-MBD efficiently.
